# Supplementary material for: A Single-Cell Atlas of the Substantia Nigra Reveals Therapeutic Effects of Icaritin in a Rat Model of Parkinson’s Disease
Source: Antioxidants (Basel). 2024 Sep 30;13(10):1183. doi: 10.3390/antiox13101183 (PMC11505506; doi:10.3390/antiox13101183)
Supplement: Supplementary file 1 [file antioxidants-13-01183-s001.zip › Supplementary Material Figures.pdf]

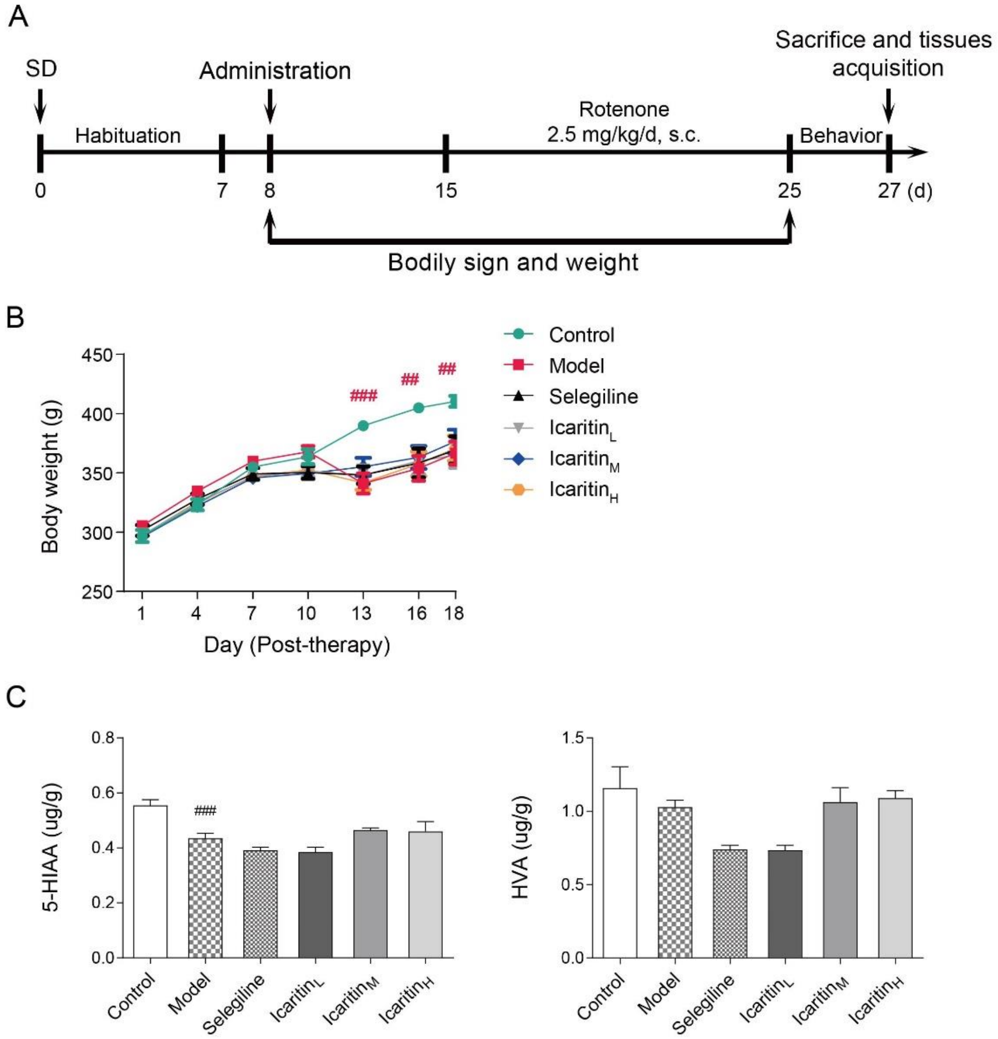

Figure S1. Establishment of a rotenone-induced PD rat model. A. Schedule of rotenone-induced PD rat treatments. B. Body weights of each group. C. HPLC analysis of HVA and 5-HIAA levels. Data are presented as the mean ± SEM. n=4-5. Control, control group; Model, PD model group; Selegiline, selegilin-treated group; Icaritin$_L$: 3.27 mg/kg icaritin-treated group; Icaritin$_M$: 6.54 mg/kg icaritin-treated group; Icaritin$_H$: 13.08 mg/kg icaritin-treated group. $^{\#\#}$ $P < 0.01$, $^{\#\#\#}$ $P < 0.001$ vs. Control group.

.

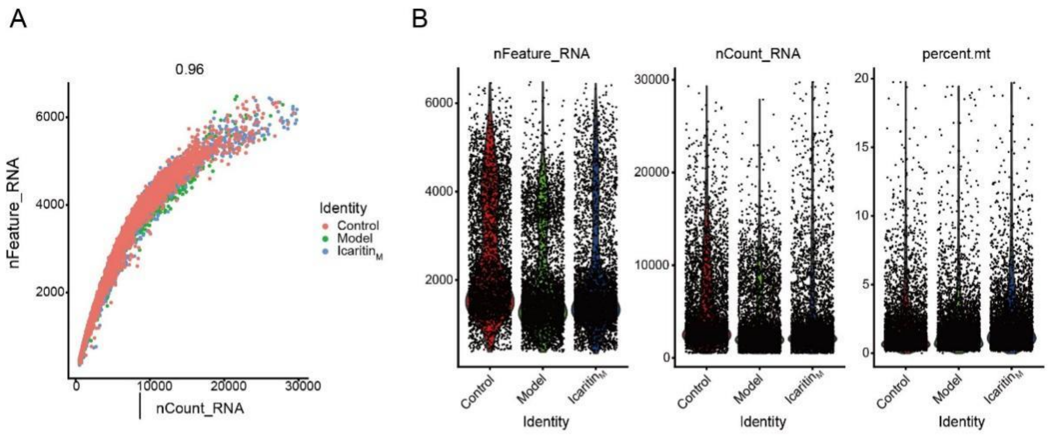

Figure S2. Quality Control (QC) plots. A. The number left the figure was the Pearson correlation coefficient between the number of UMI and genes in all three samples. The closer the correlation coefficient was to 1, the stronger the correlation was. B. Violin plots showing the distribution of the number of genes, number of counts, and percentage of mitochondrial fraction in all three samples. To reduce the number of low-quality cells, doublet and multi-cell ratio, cells were filtered out if more than 2,0000 counts, less than 500 genes were detected, and the percentage of mitochondrial gene counts higher than 25%.

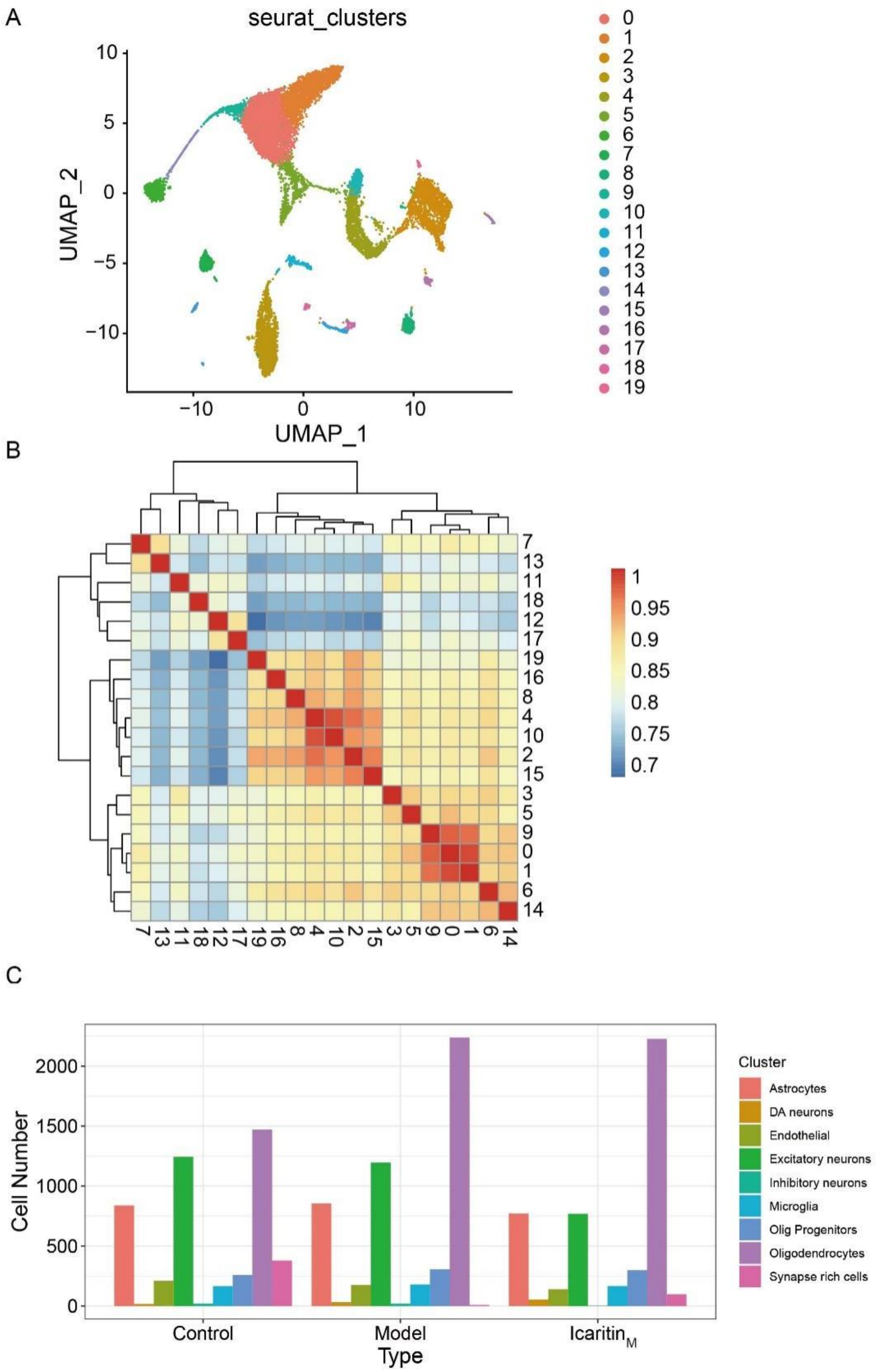

Figure S3. Cell composition of all groups. A. All clusters were obtained by single-cell analysis in a UAMP plot. The 15 cell types were identified with classic marker genes by CellRanger software. B. The main cell types are distributed evenly in general across three groups. C. The cell numbers of all subtypes in three groups.

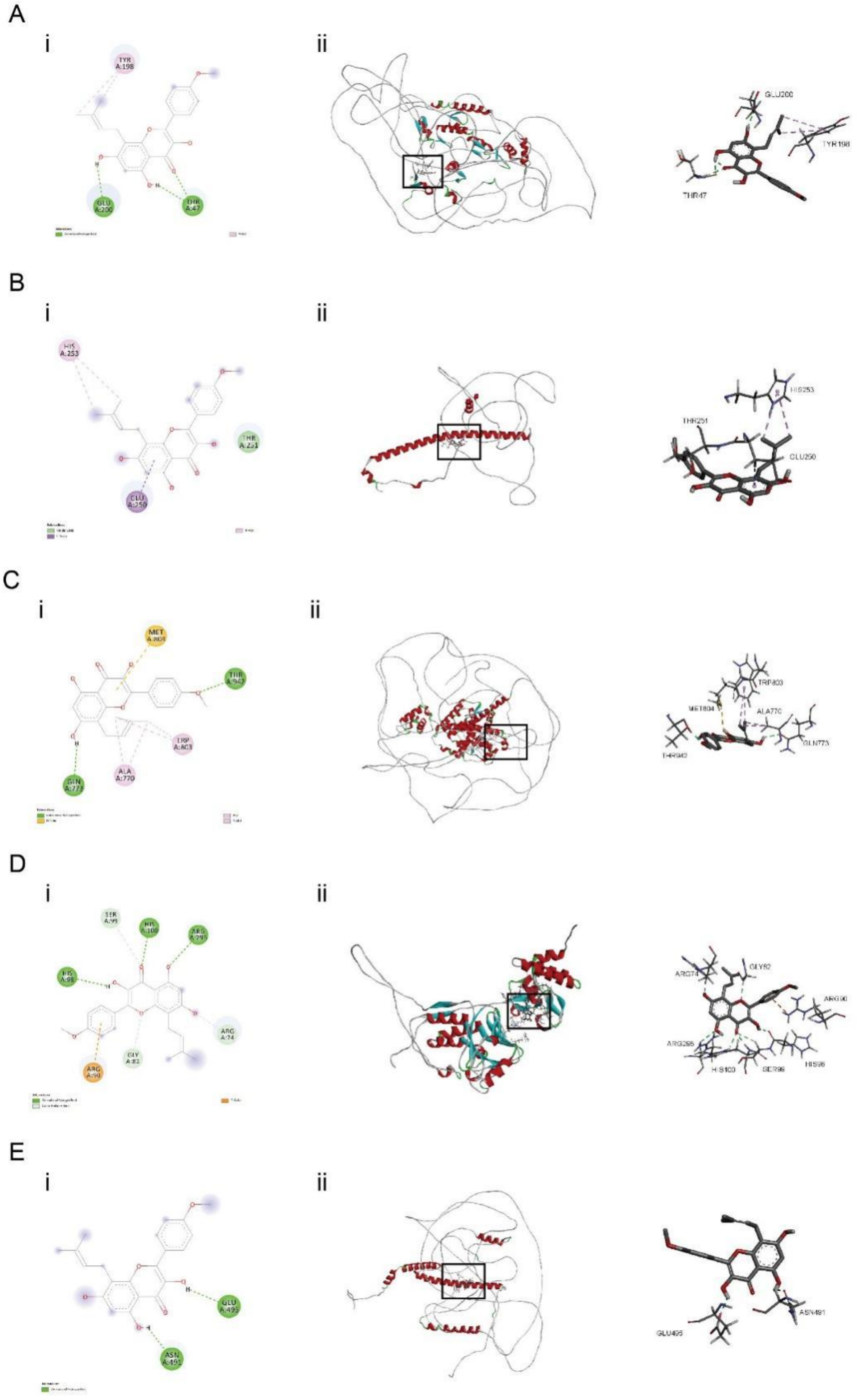

Figure S4. Icaritin bound to transcription factors (TFs) in the SRCs. A-E. Molecular docking results of icaritin's on BCL11A (A), CEBPB (B), NR3C2 (C), SMAD3 (D), and TCF4 (E). The red (i) and dark molecule (ii) is icaritin..

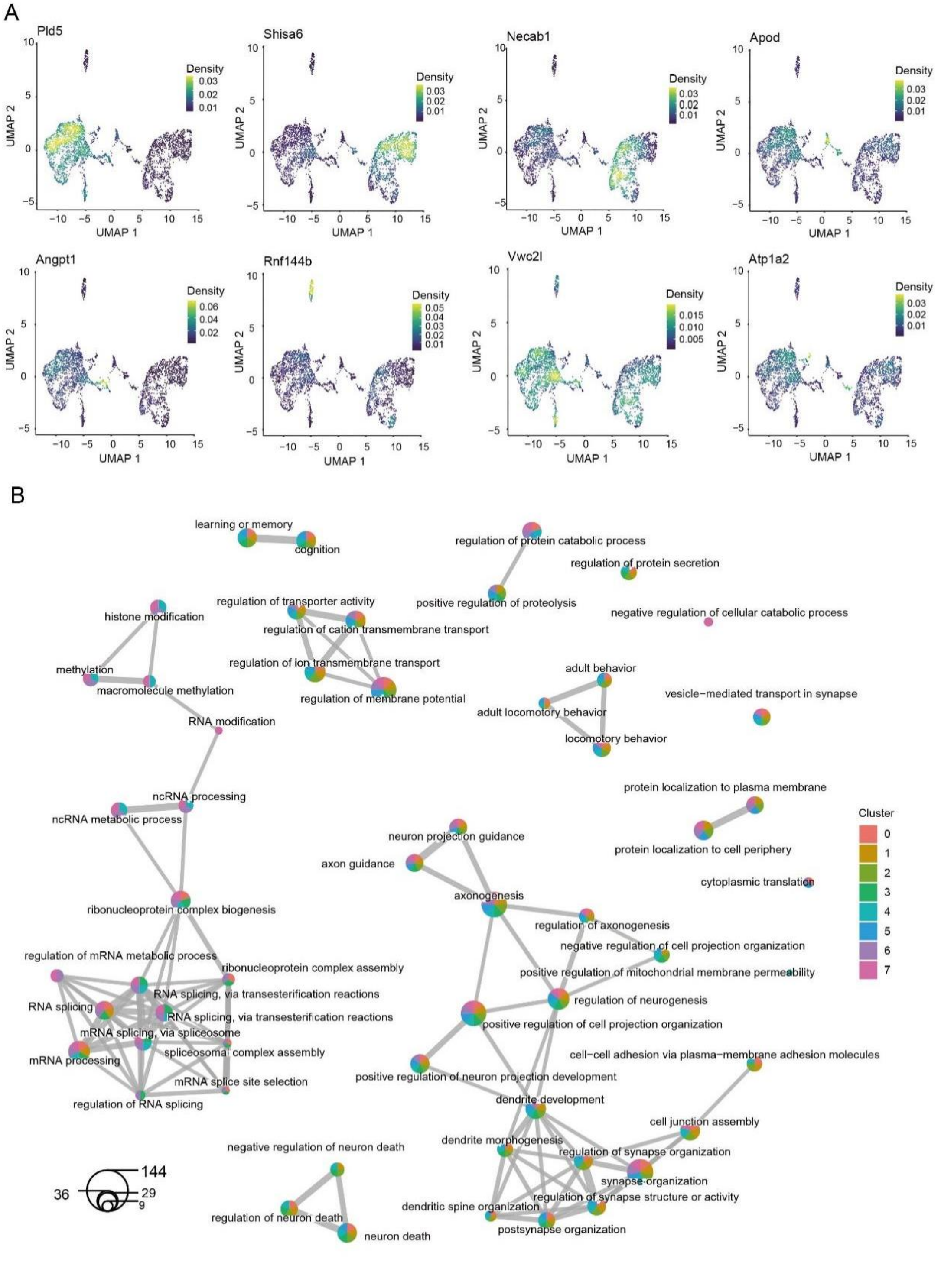

Figure S5. Marker genes of eight subclusters in neuron and GO analysis in each indicated gene clusters. A. Subcluster cell markers were used to label clusters by cell identity as represented in the UMAP plot. Data are colored according to expression levels and the legend is labeled in log scale. B. GO analysis showing enriched terms in each indicated gene clusters using the differentially expressed genes (DEGs) among the cell population of the Icaritin samples compared to Model.

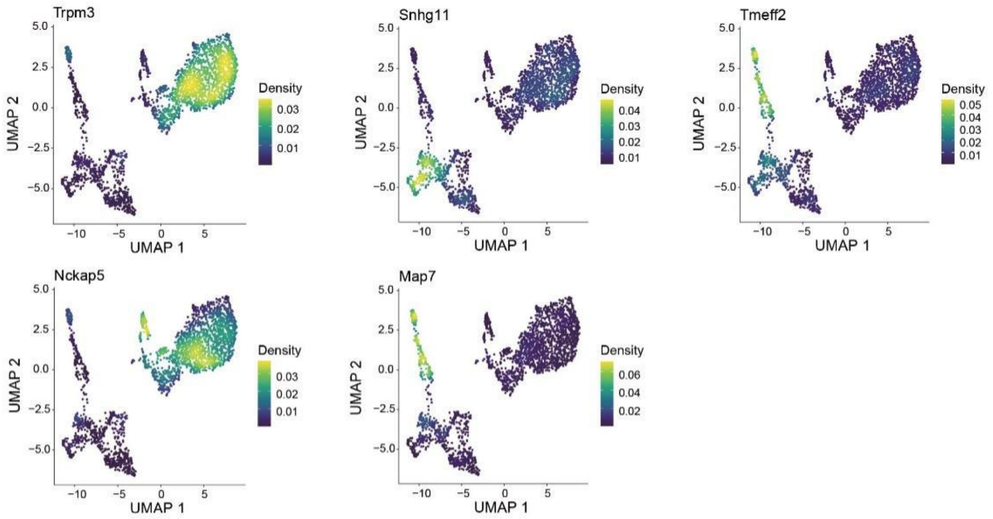

Figure S6. Marker genes of five subclusters in astrocytes. Subcluster cell markers were used to label clusters by cell identity as represented in the UMAP plot. Data are colored according to expression levels and the legend is labeled in log scale.

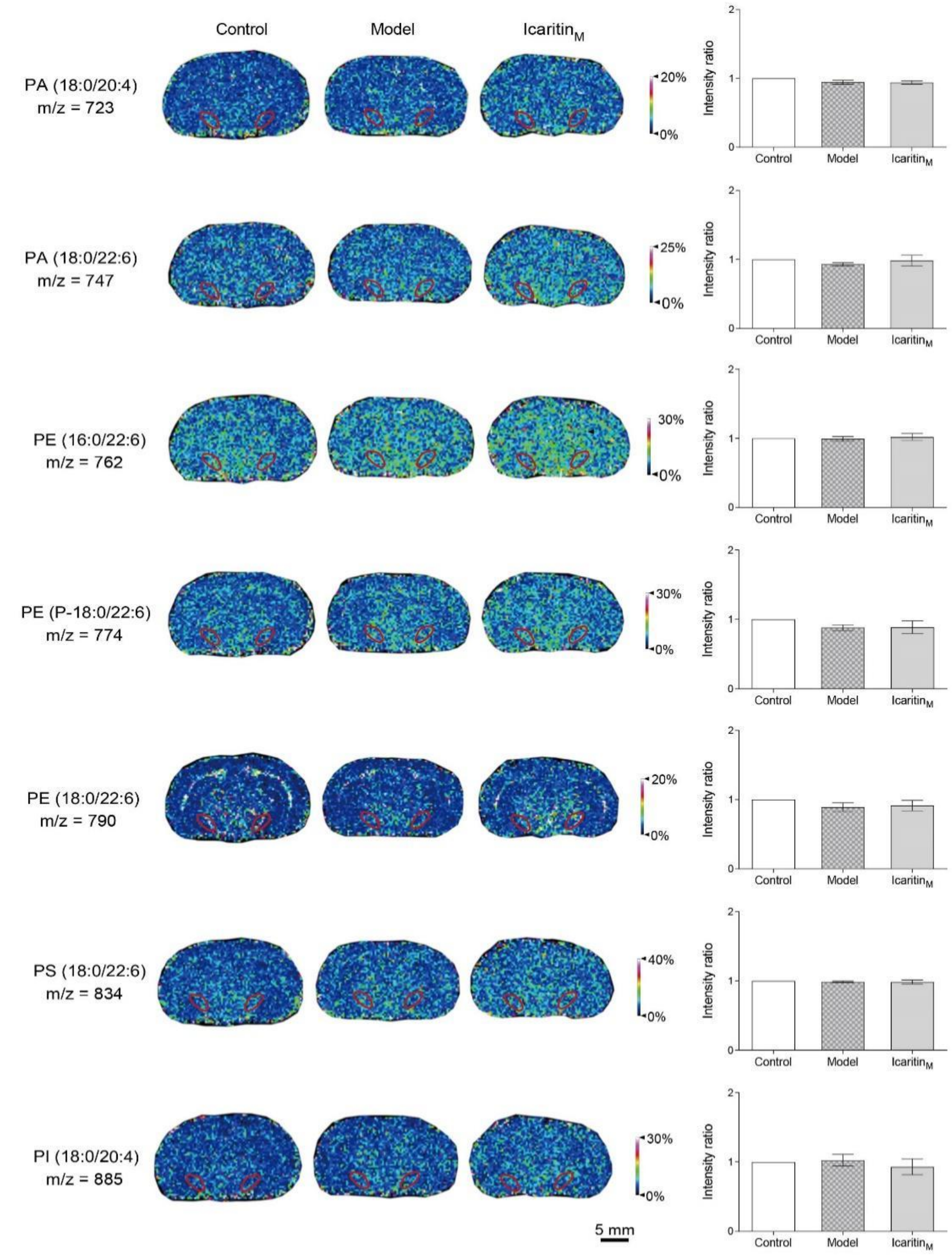

Figure S7. Icaritin had no effect on the other seven phospholipids in the substantia nigra of PD rats. *In situ* MALDI–MSI of PA (18:0/20:4), PA (18:0/22:6), PE (16:0/22:6), PE (P-18:0/22:6), PE (18:0/22:6), PS (18:0/22:6), and PI (18:0/20:4). Spatial resolution = 200 μm; scale bar = 5 mm. m/z: mass-to-charge ratio. The area selected by the red line is substantia nigra. PA, phosphatidic acid. PE, phosphatidylethanolamine. PS, Phosphatidylserine. PI, Phosphatidylinositol. Control, control group; Model, PD model group; Icaritin$_M$, 6.54 mg/kg icaritin-treated group. Data are presented as mean ± SEM; n = 3 per group. $^{\#}$ *P* < 0.05, $^{\#\#}$ *P*< 0.01 vs. Control group; ** *P* < 0.01 vs. Model group.

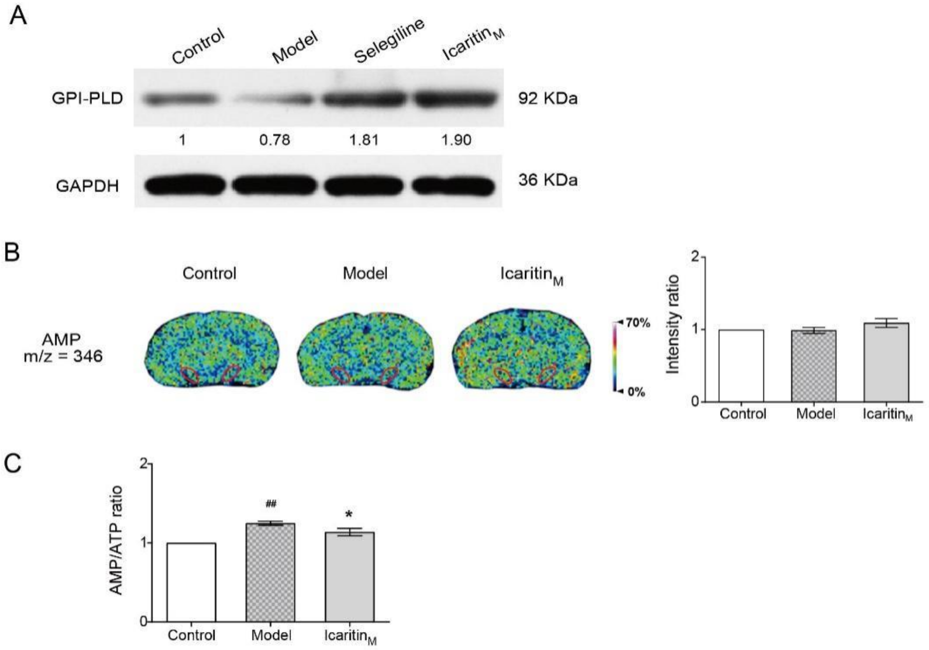

Figure S8. Icaritin regulated GPI-PLD and AMP/ATP ratio in the PD rats. A. Western blot analysis of GPI-PLD in the midbrain of rats treated with rotenone in the presence or absence of icaritin. Band intensity of the control group was set to 1, and relative values of other groups were calculated, presented under the blots. n = 3 per group. B. *In situ* MALDI–MSI of AMP. Spatial resolution = 200 μm; scale bar = 5 mm. m/z: mass-to-charge ratio. C. Icaritin regulated AMP/ATP ratio in the substantia nigra of PD rats. Control, control group; Model, PD model group; Selegiline, selegilin-treated group; $Icaritin_M$, 6.54 mg/kg icaritin-treated group. Data are presented as mean ± SEM; n = 3 per group. $^{\#\#}$ *P*< 0.01 vs. Control group; * *P* < 0.05 vs. Model group.

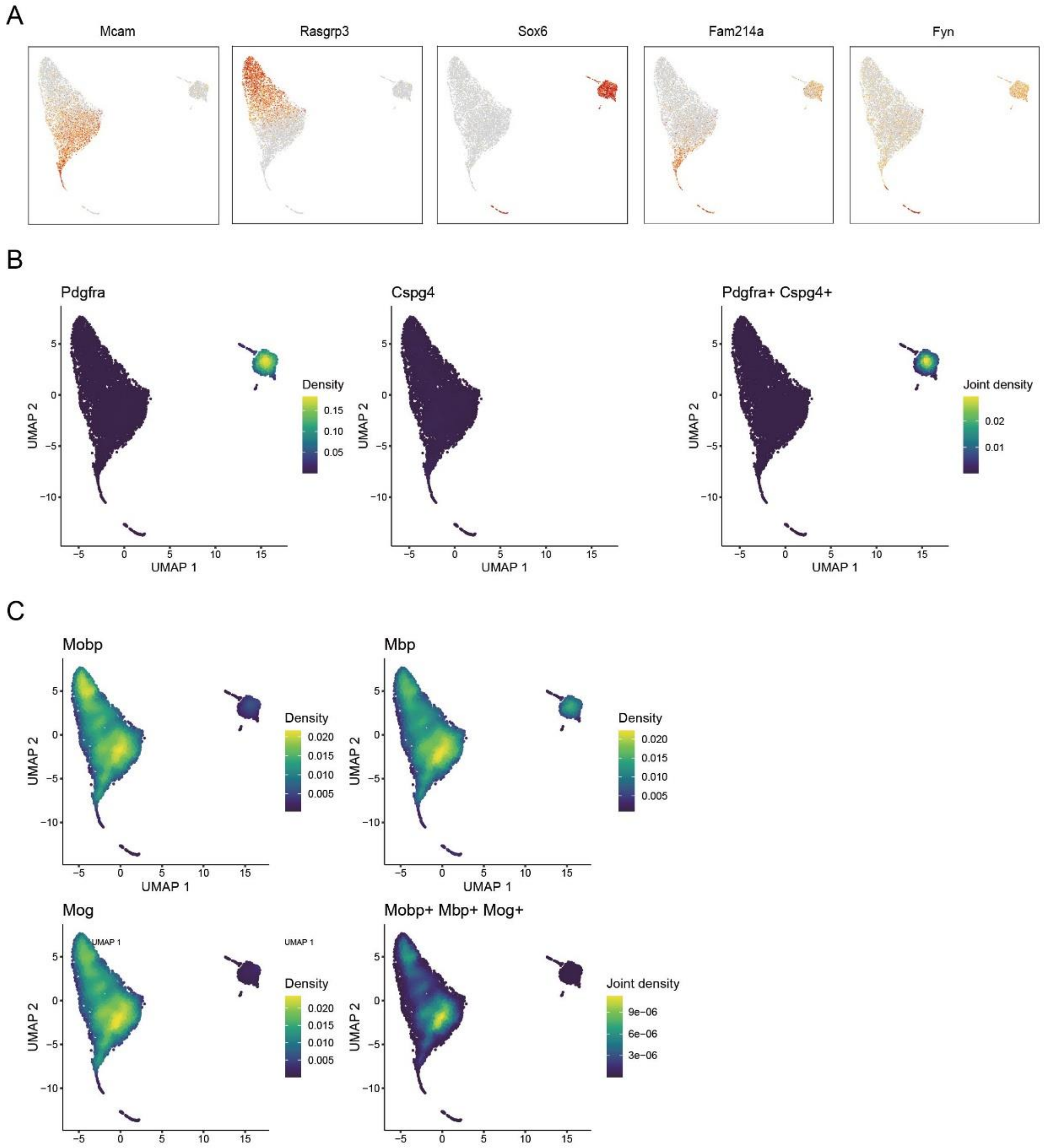

Figure S9. Marker genes of oligodendrocytes and oligodendrocyte progenitor cells. A. Subcluster cell markers were used to label clusters by cell identity as represented in the UMAP plot. Data are colored according to expression levels and the legend is labeled in log scale. B, C. Cell density plots showing combined markers gene of oligodendrocytes (B) and oligodendrocyte progenitor cells (C).

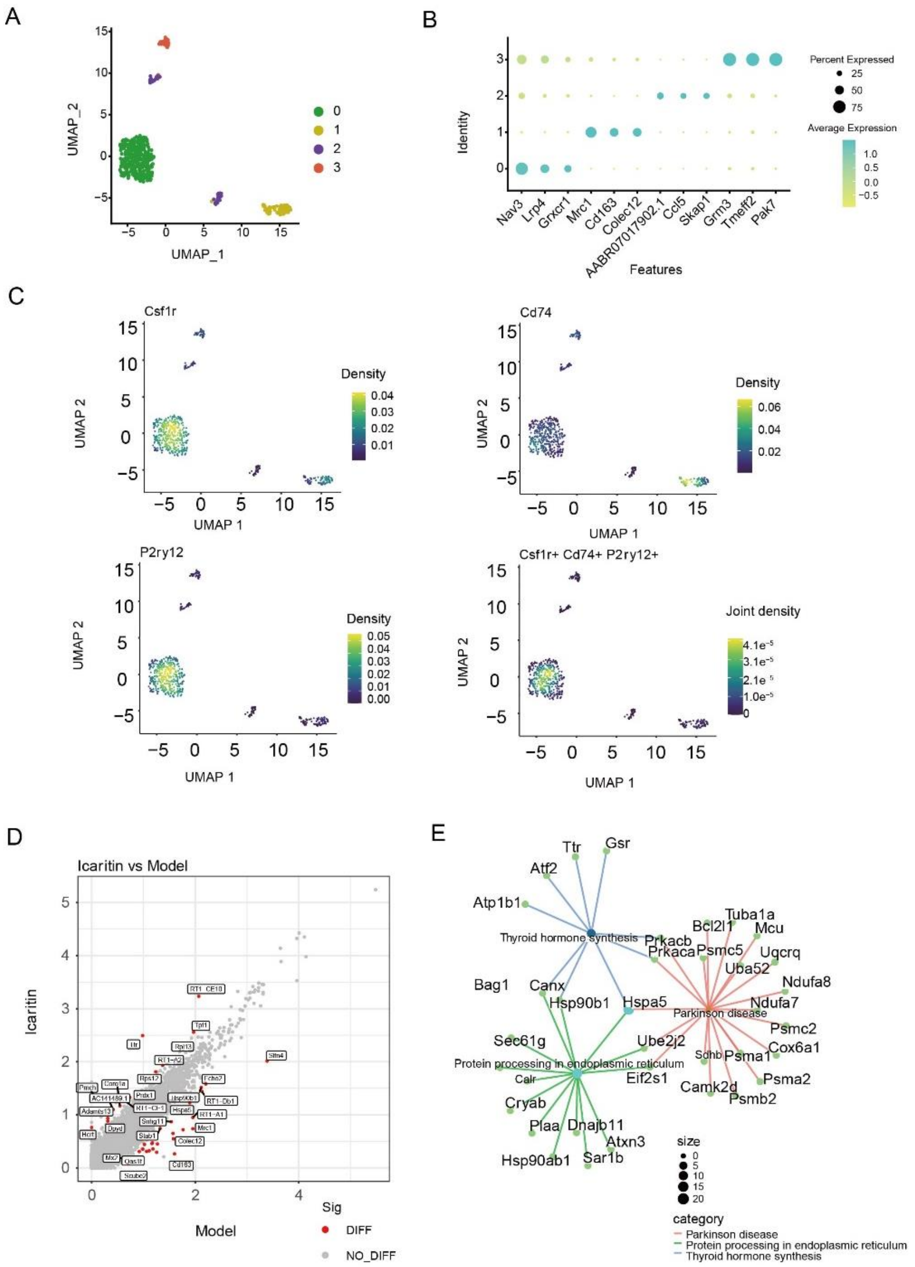

Figure S10. Microglia cells in the substantia nigra of PD rats. A. Four main Microglia cell (510 nuclei) subclusters were identified by UMAP analysis. B. Dot plots showing the 12 signature gene expressions across the 4 cellular clusters. C. Subcluster cell markers were used to label clusters by cell identity as represented in the UMAP plot. Data are colored according to expression levels and the legend is labeled in log scale. D. The scatter plot of the DEGs between Microglia cells from Icaritin samples versus Model. The top 10 DEGs in each comparison were labeled in red. E. Cnetplot showing the top most enriched GO terms in significantly differentially expressed genes among the cell population of the icaritin group compared to the model group. Hypergeometric overrepresentation test, Benjamin–Hochberg multiple testing correction.

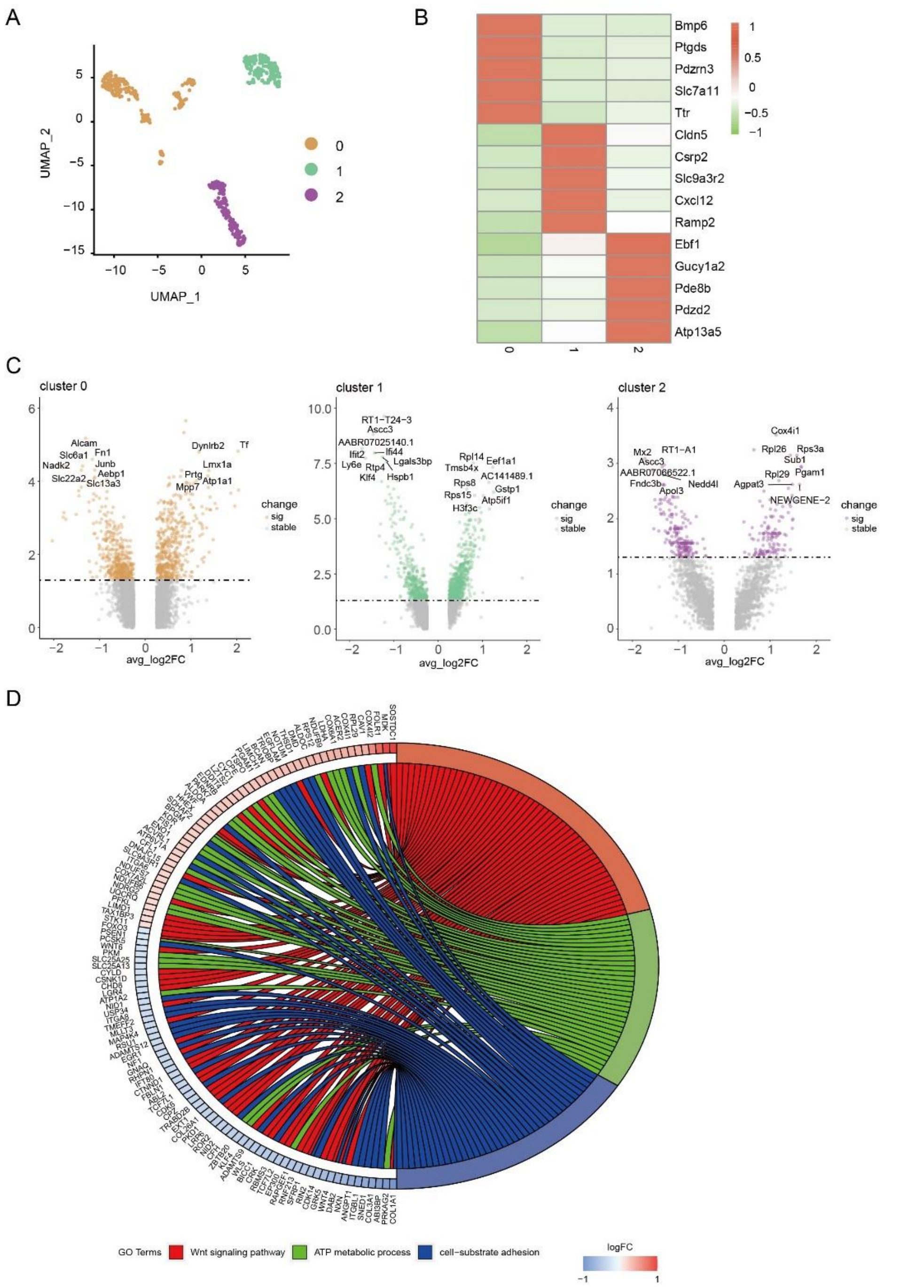

Figure S11. Endothelial cells in the substantia nigra of PD rats. A. Four main subclusters endothelial cells (522 nuclei) were identified by UMAP analysis. B. The heatmap of the average expression of top 5 DEGs among three subclusters of Endothelial cells. The color legend indicates normalized gene expression levels among the subclusters.C. Volcano plot showing differentially expressed genes between the icaritin group and the model group among three subclusters. D. A circle plot showing the top three changed pathways and the corresponding top regulated genes according to the enrichment analysis based on DEGs in Endothelial cells.

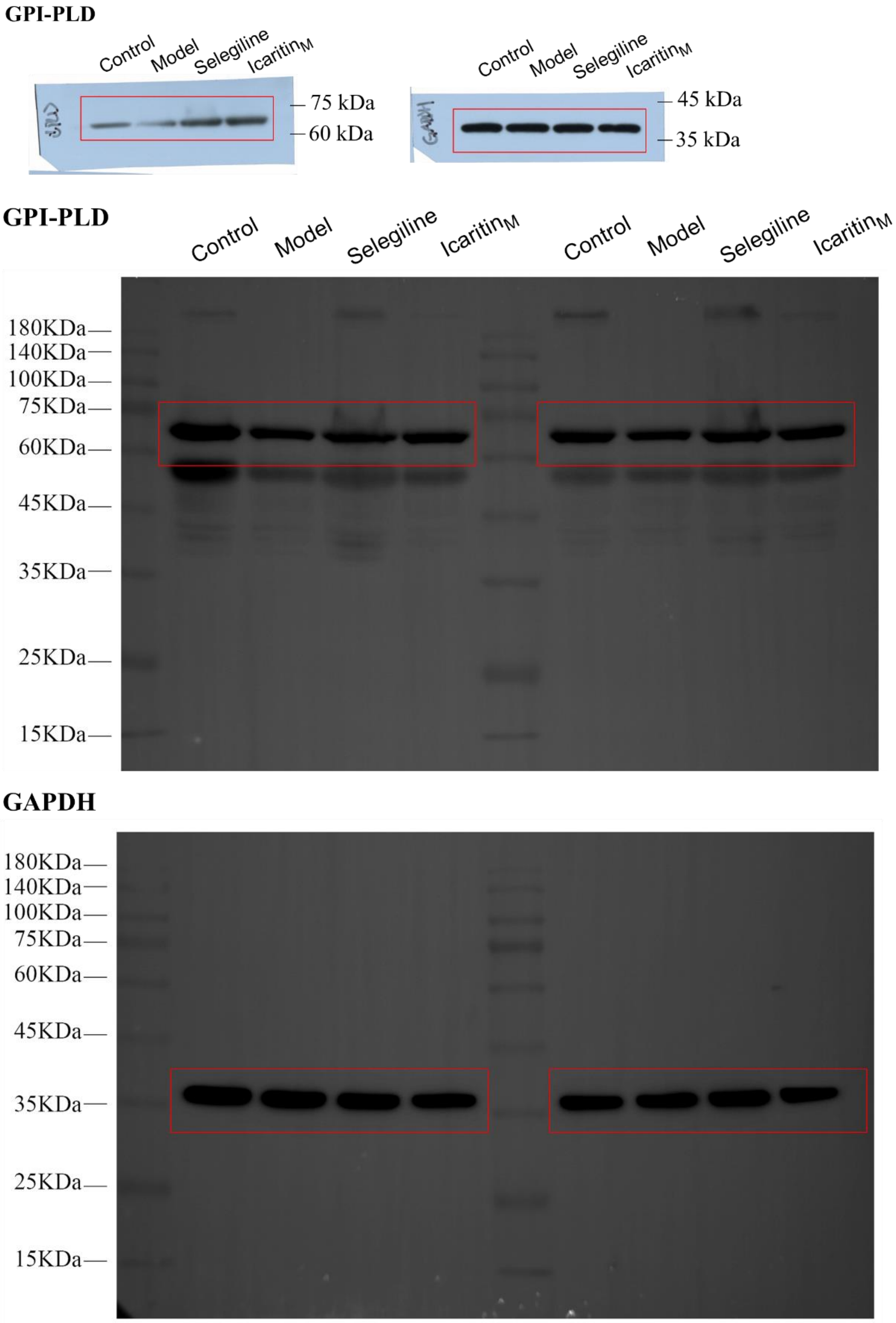

Figure S12. Full gel scans for western blot. Above: the gel scan for Figure S8A. Below: the other two independent experiments with replications. C. Icaritin regulated AMP/ATP ratio in the substantia nigra of PD rats. Control, control group; Model, PD model group; Selegiline, selegilin-treated group; $Icaritin_M$, 6.54 mg/kg icaritin-treated group.
